# The First Complete 3D Reconstruction of a Spanish Fly Primary Larva (*Lytta vesicatoria*, Meloidae, Coleoptera)

**DOI:** 10.1371/journal.pone.0052511

**Published:** 2012-12-26

**Authors:** Si-Qin Ge, Benjamin Wipfler, Hans Pohl, Yi Hua, Adam Ślipiński, Xing-Ke Yang, Rolf Georg Beutel

**Affiliations:** 1 Key Laboratory of Zoological Systematics and Evolution, Institute of Zoology, Chinese Academy of Sciences, Beijing, China; 2 Entomology Group, Institut für Spezielle Zoologie and Evolutionsbiologie mit Phyletischem Museum, Friedrich-Schiller-Universität Jena, Jena, Germany; 3 CSIRO Ecosystem Sciences, Australian National Insect Collection, Canberra, Australia; Sars International Centre for Marine Molecular Biology, Norway

## Abstract

The first detailed anatomical study of a primary larva of Meloidae is presented. Thereby techniques such as three-dimensional reconstructions, microtome sections, SEM (scanning electronic microscopy) and CLSM (confocal laser scanning microscopy) are applied. The structural features are discussed in the context of phylogeny, but also possible correlations with parasitism, phoresy and miniaturisation. The triungulin first instar larva is likely an apomorphy of Meloidae excl. Eleticinae and linked with a specialisation on acridoid eggs or larvae and provisions of bees. The campodeid body shape of *Lytta* and Meloinae is a groundplan feature of Meloidae, whereas a navicular body is an autapomorphy of the generally phoretic larvae of Nemognathinae. Head structures of *Lytta* and features of the postcephalic body are largely plesiomorphic. The musculature of the head is only moderately simplified while the one of the postcephalic body is well developed. Its thorax is largely characterised by plesiomorphies. The characteristics of the legs suggest phoretic habits, even though this does not apply to larvae of *Lytta*. It is conceivable that a phoretic behaviour is secondarily lost, together with some but not all morphological modifications related to it. Derived features of the abdomen of Meloidae are the complete loss of the fixed urogomphi (also missing in Rhipiphoridae and other related groups) and the presence of one or two conspicuous caudal bristles. Only few features of *Lytta* are shared with the parasitic larvae of Rhipiphoridae and Strepsiptera. These characteristics, which are possibly linked with specialised life habits, have obviously evolved independently. Miniaturisation effects are minimal in the larvae of *Lytta*.

## Introduction

*Lytta vesicatoria* (Linnaeus) is a charismatic species of the medium sized tenebrionoid family Meloidae, which comprises ca. 3000 spp. worldwide (e.g. [1]; [2]). The Meloidae group occurs on all continents with the exception of Antarctica but is absent from New Zealand and most Polynesian islands. *Lytta vesicatoria* is a fairly large and conspicuous beetle with a metallic emerald-green coloration. It occurs mainly in the Mediterranean area but occasionally it is also collected in Central or Eastern Europe and Asia The adults are usually found exposed on its food plants, especially *Fraxinus excelsior* (Common Ash) and *F. ornus* (Manna Ash), but also on poplar, elderberry, lilac, olive and others. Like in other “blister beetles” (Meloidae) males produce cantharidin, a vesicating substance functioning as a very efficient and powerful defensive natural product [2]. Cantharidin is transmitted during copulation and placed on the egg surface by the females. The powerful terpenoid can cause blisters and seriously affect the gastrointestinal and urinary tract, and also the kidneys (e.g. [3]). Historically it is well known as an aphrodisiac, supposedly used by prominent figures like for instance Livia, the wife of the Roman emperor Augustus, the German Emperor Henry IV, or the Marquis de Sade. Irritation of urethral passages and inflammation of the genitalia can lead to painful priapism. In the 19^th^ century cantharidin was one of the most poisonous known substances and only as little as 10 mg can be lethal for humans. In ancient China, the beetles were mixed with excrement, arsenic and wolfsbane to create the world's first recorded stink bomb.

Another remarkable feature of *Lytta* and the other meloids (with the possible exception of Eleticinae; [2]) is a specific type of parasitism, linked with hypermetamorphosis, and miniaturised triungulin first instar larvae. The tringulins of many meloid groups (Meloinae partim, Nemognathinae [excl. *Stenodera*] are phoretic and attach to their bee hosts as they visit flowers [2]; [4]. This is not the case in *Lytta* [5] even though they seem to be well adapted for such a behaviour (e.g., triungulin legs). The hypermetamorphic development passes through eight immature stages, the tringulin, the first grub (instars II–V), the coarctate (instar VI), the second grub (instar VII), and the pupa. After attaining the host’s nest the triungulin starts to feed on the bee’s food-stores and eventually molts [2]; [4]. The four stages of the relatively immobile but feeding scarabaeiform first grub is followed by the inactive coarctate, which is characterized by a diapause. Then follows the active but non-feeding second grub, which is morphologically similar to the first grub and finally prepares the pupal chamber.

The focus of the present study is on the agile first instar larvae, the stage which is crucial for the access to the host’s resources (or body) as it is the case in different ways in several other parasitic groups of insects (e.g., Brachininae [Carabidae], Rhipiphoridae, Passandridae, Bothrideridae, Strepsiptera; e.g., [6]; [7]). The primary aim of our investigation is the first full documentation of the external and internal morphology of a tenebrionoid triungulin larva. Generally anatomical information on beetle larvae is relatively scarce. The head morphology of some groups is comparatively well known through a series of studies published in the two last decades (e.g. [8]; [9]; [10]; [11]), whereas internal structures of the postcephalic body are largely unknown. The presently available anatomical knowledge is restricted to a study focussed on the nervous system of the mealworm *Tenebrio molitor* [12], one on an extremely miniaturised ptiliid larva [13], and one on the larva of the corylophid *Sericoderus* [14]. The morphological findings will be discussed with respect to the unclear phylogenetic affinities of Meloidae within Tenebrionoidea, and the position of *Lytta* within the family. Another aim was to evaluate effects of miniaturisation in the small and slender triungulin larvae, following a series of recent studies on this topic using various groups of beetles but also other groups of insects (e.g. [13]; [14]; [15]; [16]). The third aim is to evaluate effects of a parasitic lifestyle on the morphology of the triungulins, also focussing on parallelisms with other parasitic groups with hypermetamorphosis, such as for instance Rhipiphoridae or Strepsiptera. Possible relationships between the endoparasitic Strepsiptera and polyphagan groups have been suggested by the celebrated coleopterist R. A. Crowson (e.g., [6]: “Stylopidae”) and were recently discussed as one possible result of analyses of an extensive molecular data set [17]. The parasitism of Strepsiptera differs fundamentally and new comprehensive molecular data demonstrate clearly that a placement of strepsipterans as a subordinate group of beetles can be excluded [18]. Nevertheless, it appeared worth-while to us to evaluate possible parallelisms. This may help to understand general patterns co-occurring with parasitic lifestyles and hypermetamorphosis in beetles and other groups of insects.

## Materials and Methods

### Examined Taxa

*Lytta vesicatoria* Linnaeus, 1^st^ instar larvae, preserved in 70% ethanol, label information: “ab ovo, 24.7.1993, Germany, Rheinland-Pfalz, Mainz-Finthen, leg. Martin Hanser”.

### Histology

Larvae of *L. vesicatoria* were embedded in Araldite, cut at 1 µm with a Microm microtome (HM 360), and stained with toluidine blue. The serial sections were photographed with the analySIS**®** system.

### Three-dimensional Reconstruction (3D)

Cross section series were used for 3-dimensional reconstructions. The images were aligned with Amira 5.1 (Visage Imaging). Based on the obtained image stacks, structures of the larvae were reconstructed with Amira 5.1. The data files were then transferred to Maya 2011 (Autodesk) in order to use the smoothing function, the specific display, and render options of this software. Final figures were prepared with Photoshop CS5 (Adobe) and Illustrator CS5 (Adobe).

### Scanning Electronic Microscopy (SEM)

Specimens were transferred to 100% ethanol, then dried at the critical point (Emitech K850 critical point dryer) and subsequently sputter-coated (Emitech K500). Microscopy was performed on a Philips XL30 ESEM using a special specimen holder [19].

### Confocal Laser Scanning Microscopy (CLSM)

For CLSM specimens were mounted between cover glass and glass slide in a drop of ethanol. Image stacks were created with a Zeiss LSM 510 using the auto fluorescence at 488 nm of argon laser.

### Muscular Nomenclature

The muscular terminology follows Wipfler et al. [20] and v. Kéler [21] (in bracelets) for the head and v. Kéler [21] for the abdomen. The thoracic muscles are consecutively numbered and do not follow any previously used nomenclature.

## Results

### 1 General Appearance and Differences between Instars

**Figure 1:**
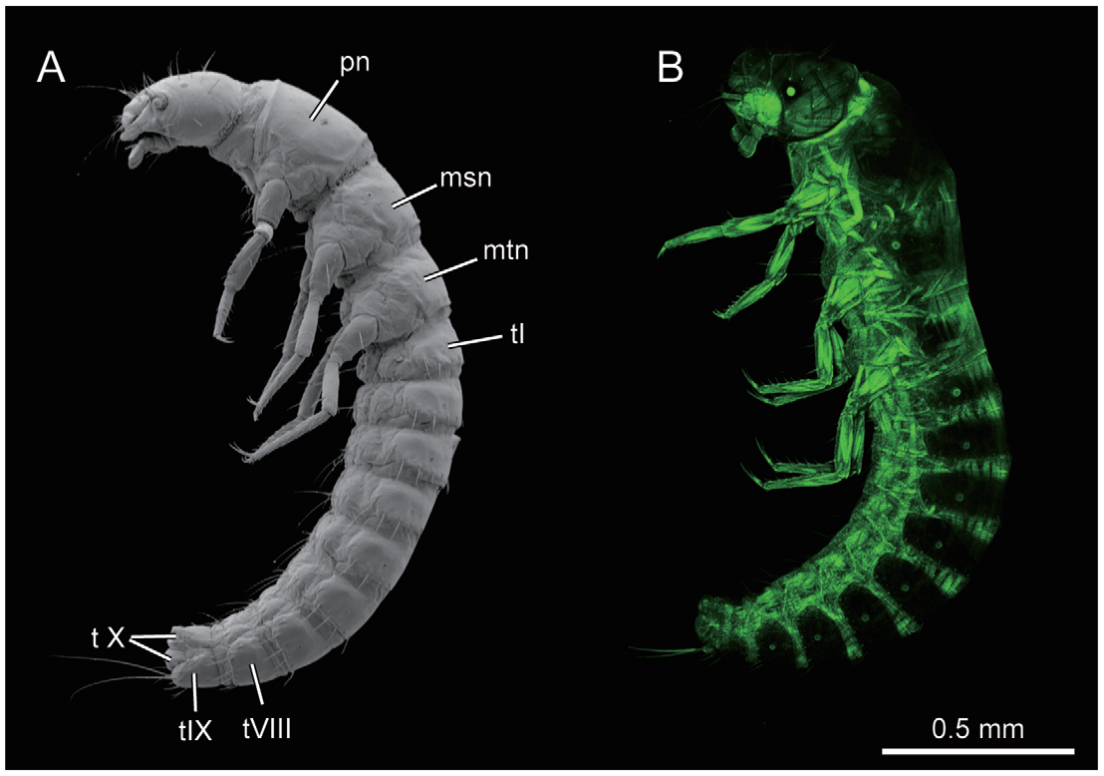
*L. vesicatoria*, habitus, lateral view. (A): SEM; (B): CLSM. Abbreviations: msn: mesonotum; mtn: metanotum; pn: pronotum; tI: abdominal tergite I; t VIII: abdominal tergite VIII; t IX: abdominal tergite IX; t X: abdominal tergite X.

The campodeiform first instar larvae are distinctly sclerotized triungulins with a slender, subparallel body and slender legs. They are slightly constricted at the metathorax and tapering at the terminal abdominal segments VIII and IX (Fig. 1). The body of second instar larvae is very lightly sclerotized and scarabaeiform. The antennae and eyes are reduced. Legs are present but shorter and less slender than in the first instar. Spiracles are present on the meso- and metathorax as well as on abdominal segments I–VIII. In the coarctate instar VI the body is more orthosomatic than in instar V. The segmentation appears indistinct. The cuticle is usually heavily sclerotized and dark. Its surface is smooth and devoid of setae. The mouthparts and legs are highly reduced and stub-like. The oral and anal openings are occluded and the gut is atrophied. Muscles are still present in the first grub stage but extremely reduced and non-functional. In the second grub (instar VII) the body is less sclerotized and the appendages less distinctly developed (2).

### 2 First Instar Larva

**Figure 2:**
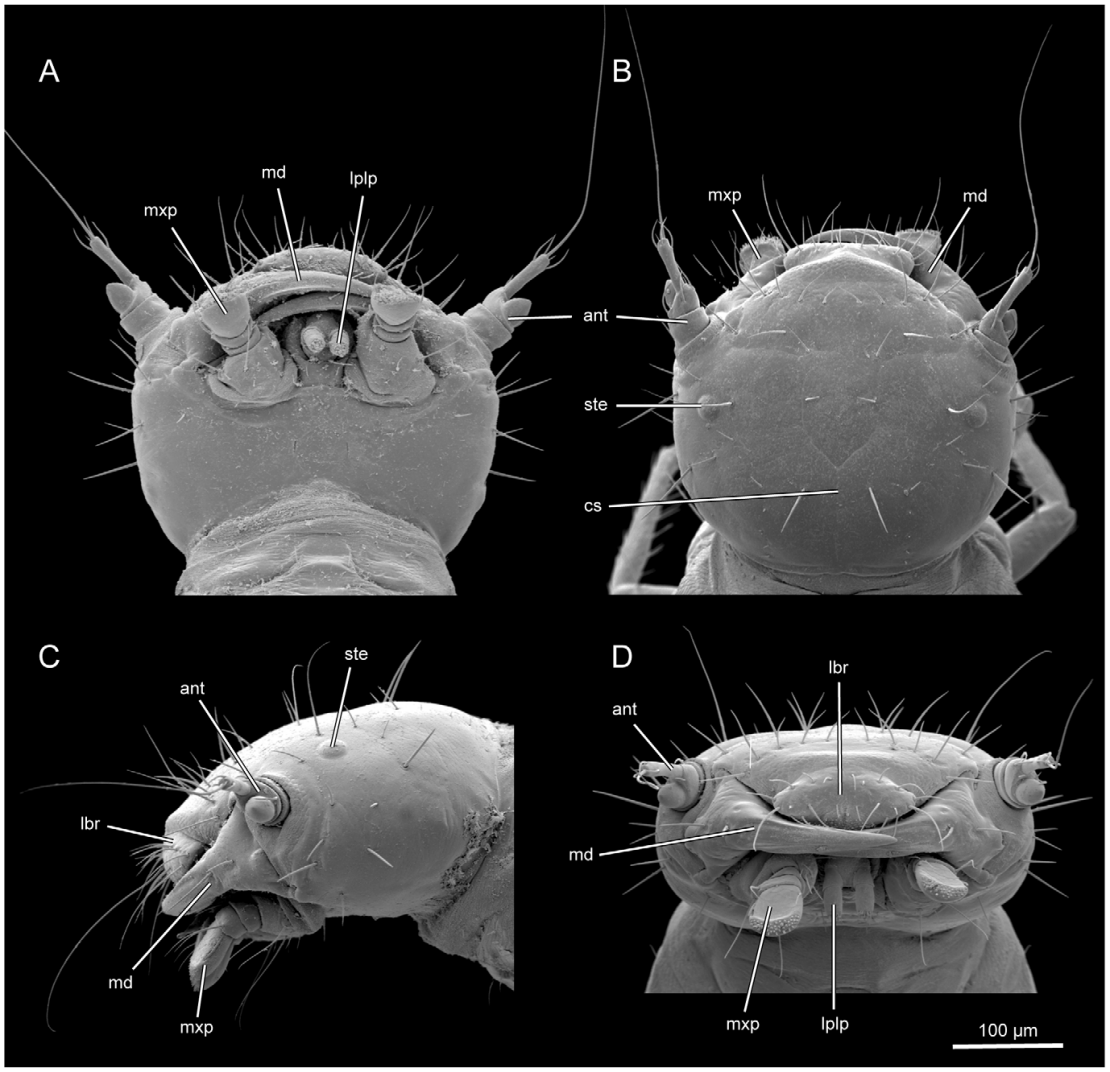
*L. vesicatoria*, head, SEM. (A): ventral view; (B): dorsal view; (C): lateral view; (D): frontal view. Abbreviations: ant: antenna; lbr: labrum; lplp: labiral palpi. md: mandible; mxp: maxillary palpi; ste: stemmata.

**Figure 3:**
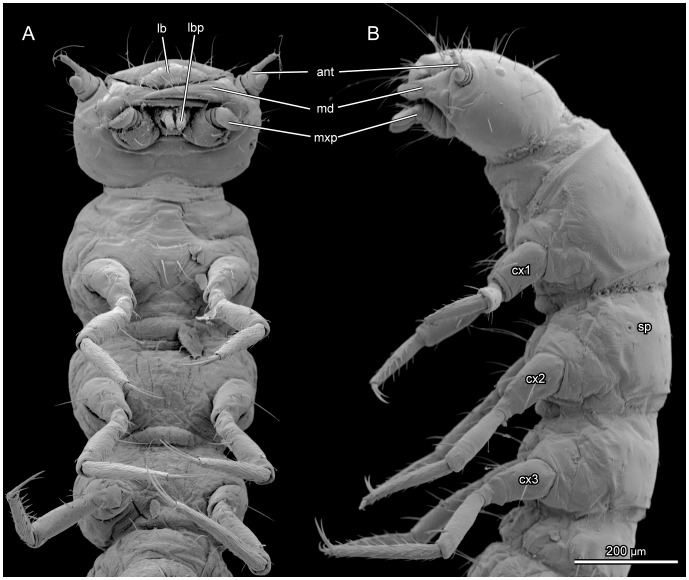
*L. vesicatoria*, head and thorax, SEM. (A) ventral view; (B) lateral view. Abbreviations: ant: antenna; cx1,2,3: pro-, meso, metacoxa; lb: labrum; lbp: labial palpus; md: mandible; mxp: maxillary palpus; sp: spiracle.

**Figure 4:**
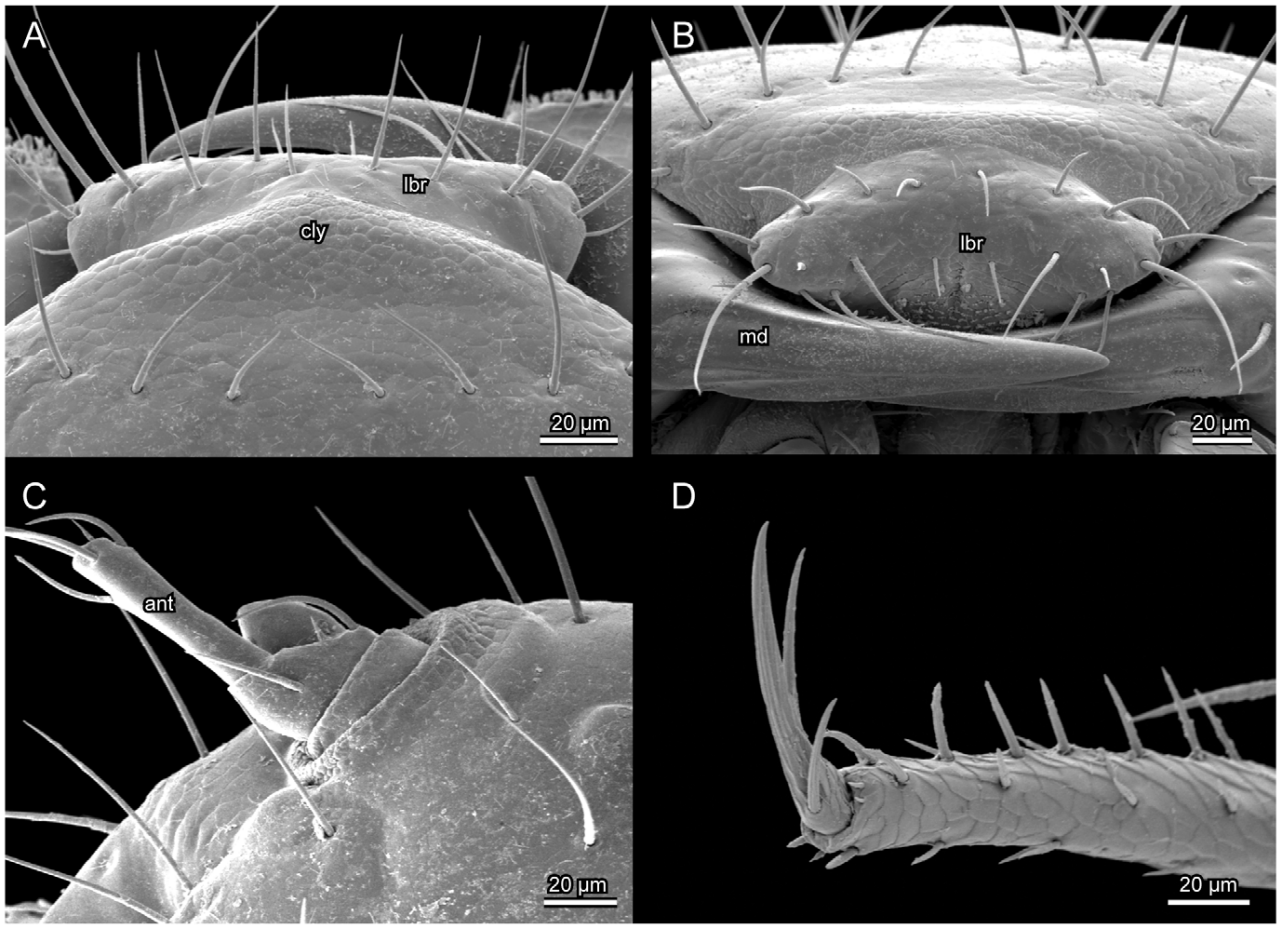
*L. vesicatoria*, SEM. (A): clypeus and labrum, dorsal view; (B):labrum and part of mandible, frontal view; (C): antenna, dorsal view; (D):tarsungulus, lateral view; Abbreviations: ant: antenna; cly: clypeus; lbr: labrum; md: mandible.

**Figure 5:**
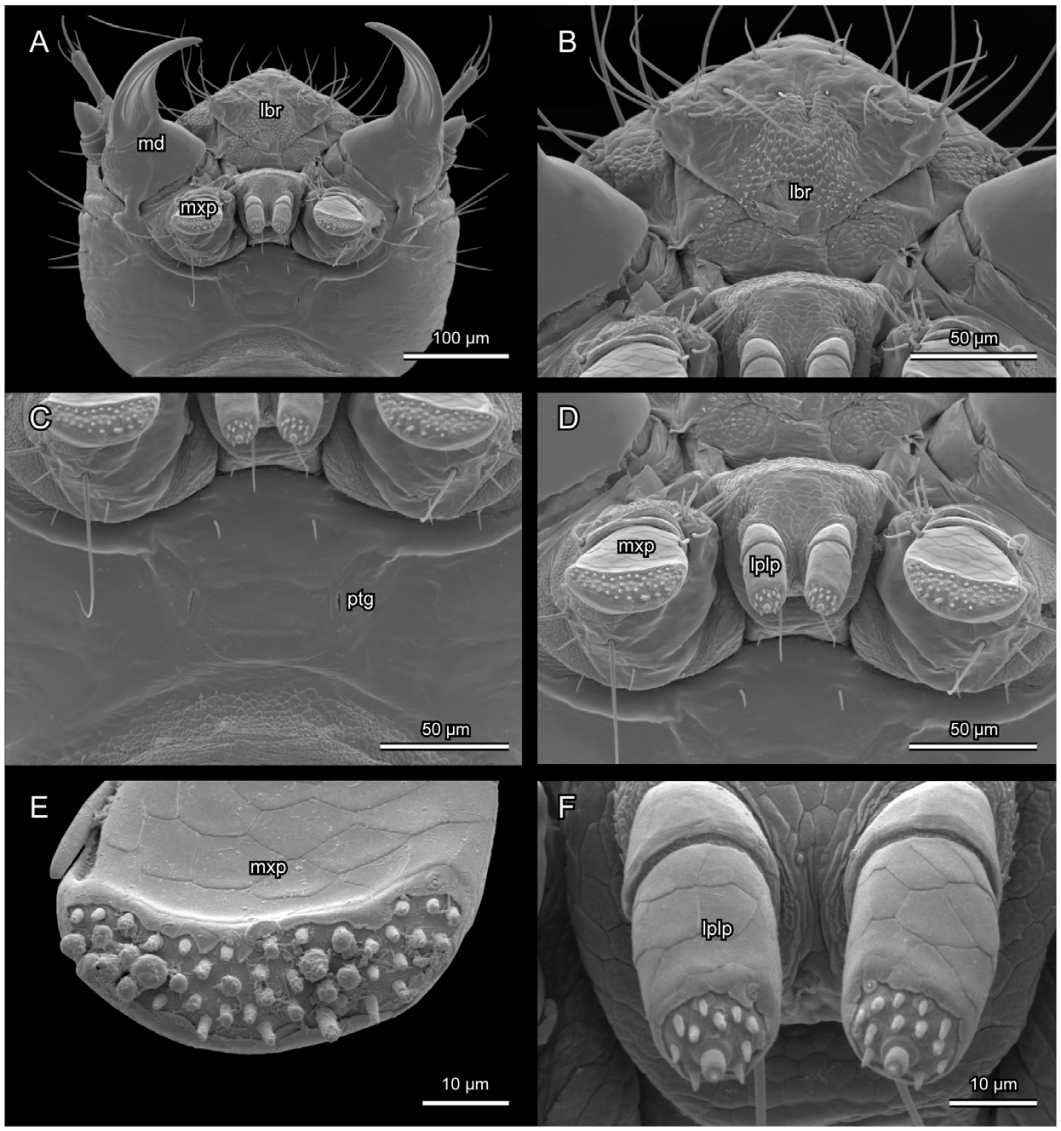
*L. vesicatoria*, ventral view, SEM. (A): head capsule, mandibles opened; (B): labral region; (C): gula region; (D): maxillae and labium; (E): maxillary palpomere 3, showing sensorial papillae; (F): labium, palpomere 2, showing spine-like sensilla. Abbreviations: lbr: labrum; lplp: labium palp; md: mandible; mxp: maxillary palp; ptg: posterior tentorial groove.

**Figure 6:**
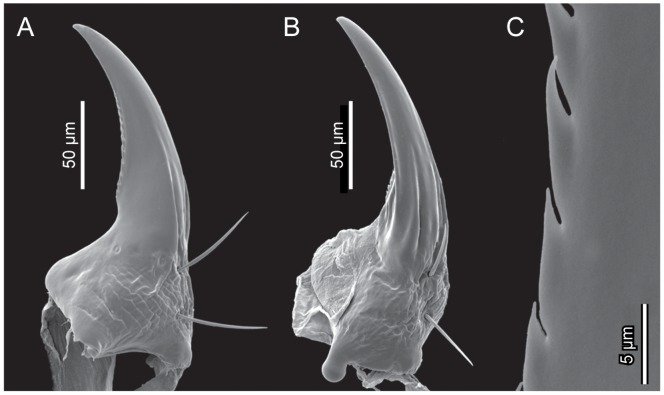
*L. vesicatoria*, mandible, SEM. (A): dorsal view; (B): lateral view; (C): distal part, showing teeth.

**Figure 7:**
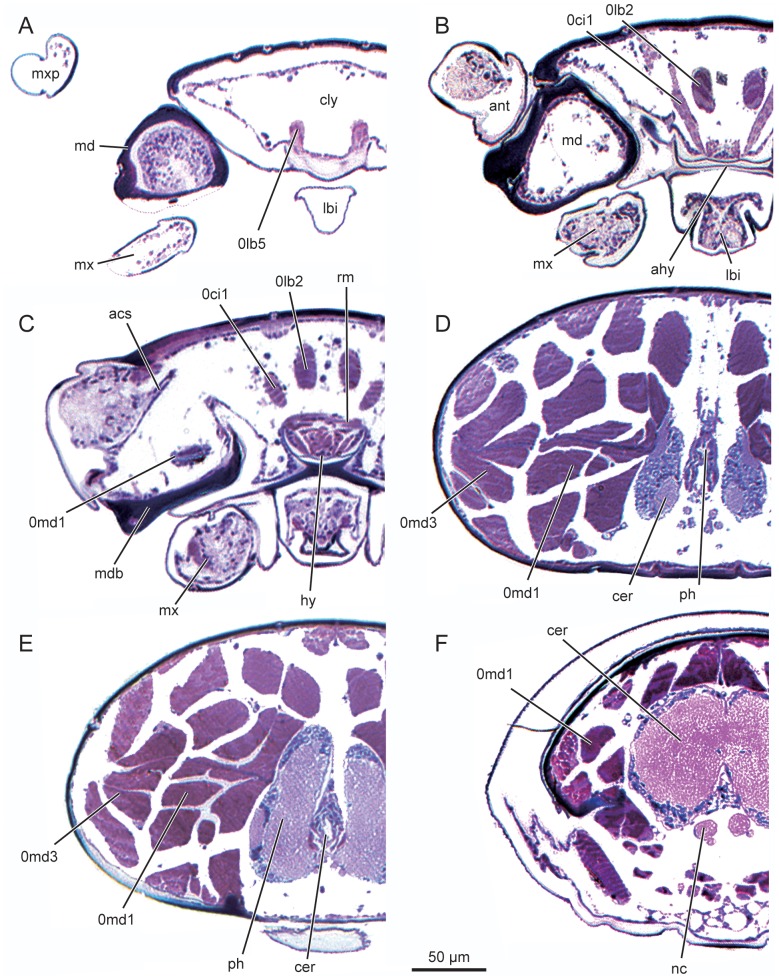
*L. vesicatoria*, head, cross section. (A–C) cross sections of the anterior head region; (D–F) cross sections of the posterior head region. Abbreviations: acs: antennal circulatory system; ahy: anterior hypopharynx; ant: antenna; cer: cerebrum; cly: clypeus; hy: hypopharynx; lbi: labium; md: mandible; mdb: base of mandible; mx: maxilla; mxp: maxillary palpus; nc: nervous cord; ph: pharynx; rm: ring muscle; 0lb2: M. frontoepipharyngalis; 0lb5: M. labroepipharyngalis; 0md1: M. craniomandibularis internus; 0md3: M. craniomandibularis externus; 0ci1: M. clypeopalatalis.

**Figure 8:**
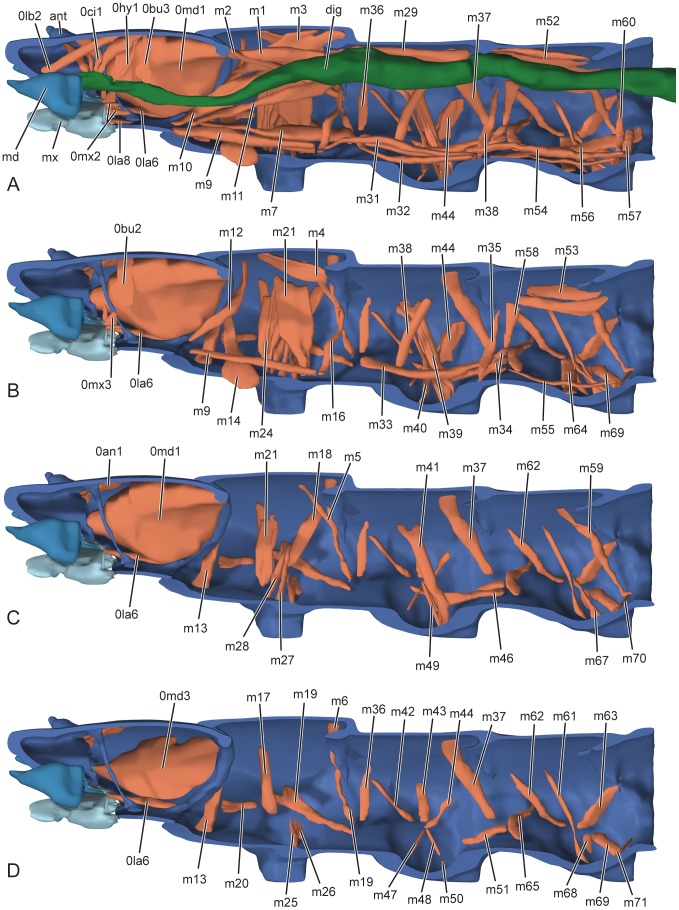
*L. vesicatoria*, head and thorax, 3D reconstructions, lateral view. Abbreviations: 0an1: M. tentorioscapalis anterior; 0bu2: M. frontobuccalis anterior; 0bu3: M. frontobuccalis posterior; 0ci1: M. clypeopalatalis; 0hy1: M. frontohypopharyngalis; 0la6: M. tentoriopraementalis superior; 0la8: M. submentopraementalis; 0md1: M. craniomandibularis internus; 0md3: M. craniomandibularis externus; 0mx2: M. craniocardinalis; 0mx3: M. tentoriocardinalis; 0lb2: M. Frontoepipharyngalis; ant: antenna; dig: digestive tract; m1–m71: thoracic musculature; md: mandible; mx: maxilla.

**Figure 9:**
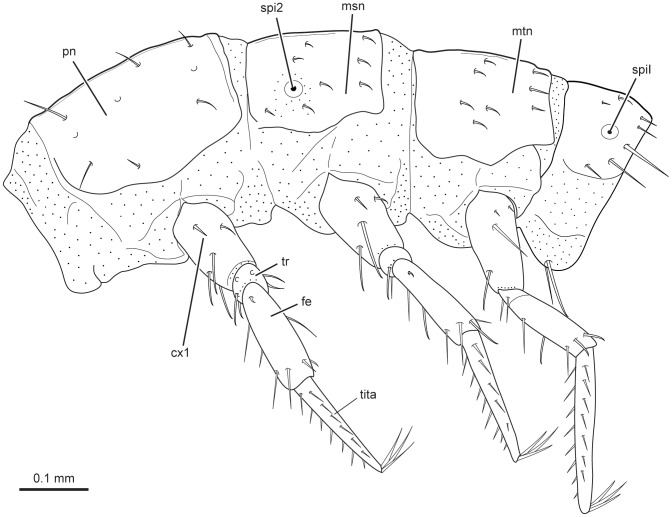
*L. vesicatoria*, thorax, lateral view, line drawing. Abbreviations: cx1: procoxa; fe: femur; msn: mesonotum; mtn: metanotum; pn: pronotum; spi2: spiracle of mesothorax; spil: spiracle of abdominal segment one; tita: tibia-tarsungulus; tr: trochanter.

**Figure 10:**
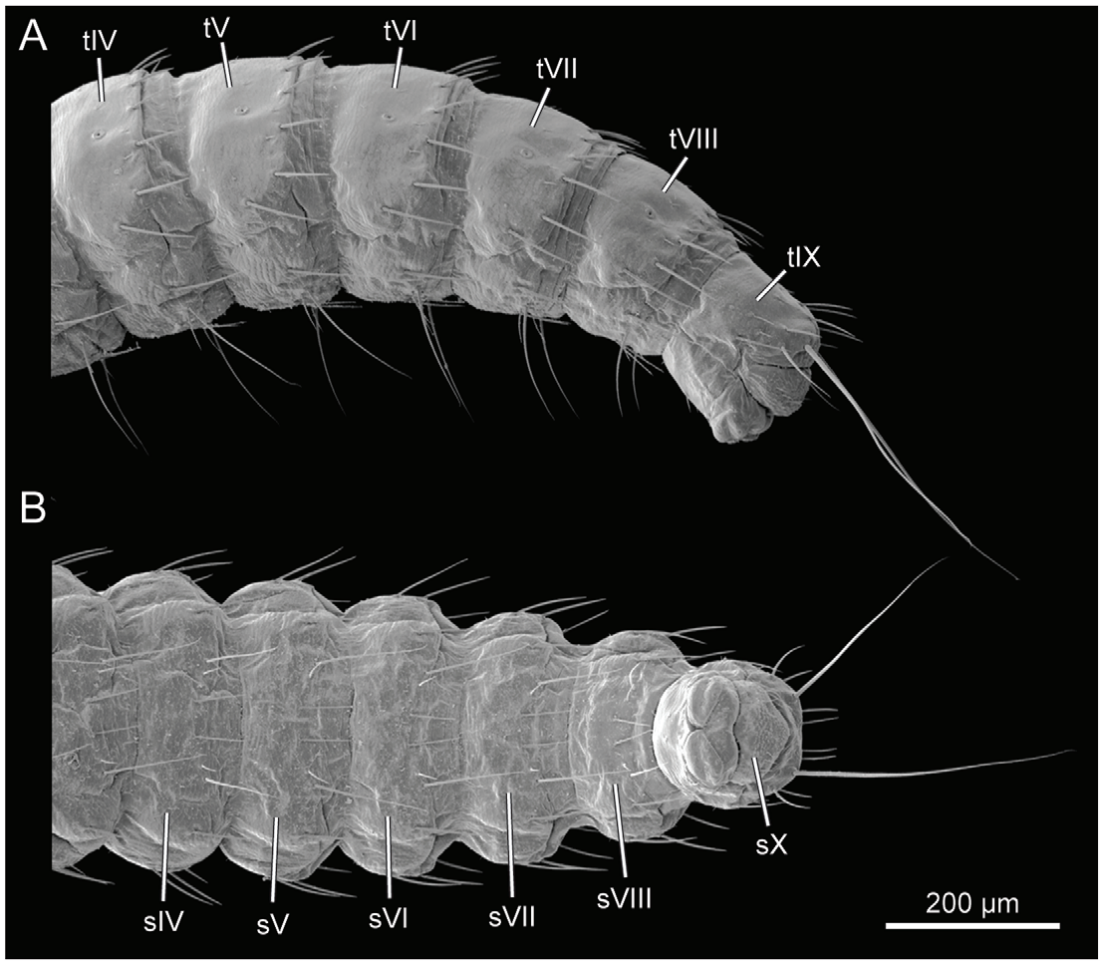
*L. vesicatoria*, abdomen, SEM. (A): lateral view; (B): ventral view. Abbraviations: sIV–X: abdominal sternites IV–X; tIV–X: abdominal tergites IV–X.

**Figure 11:**
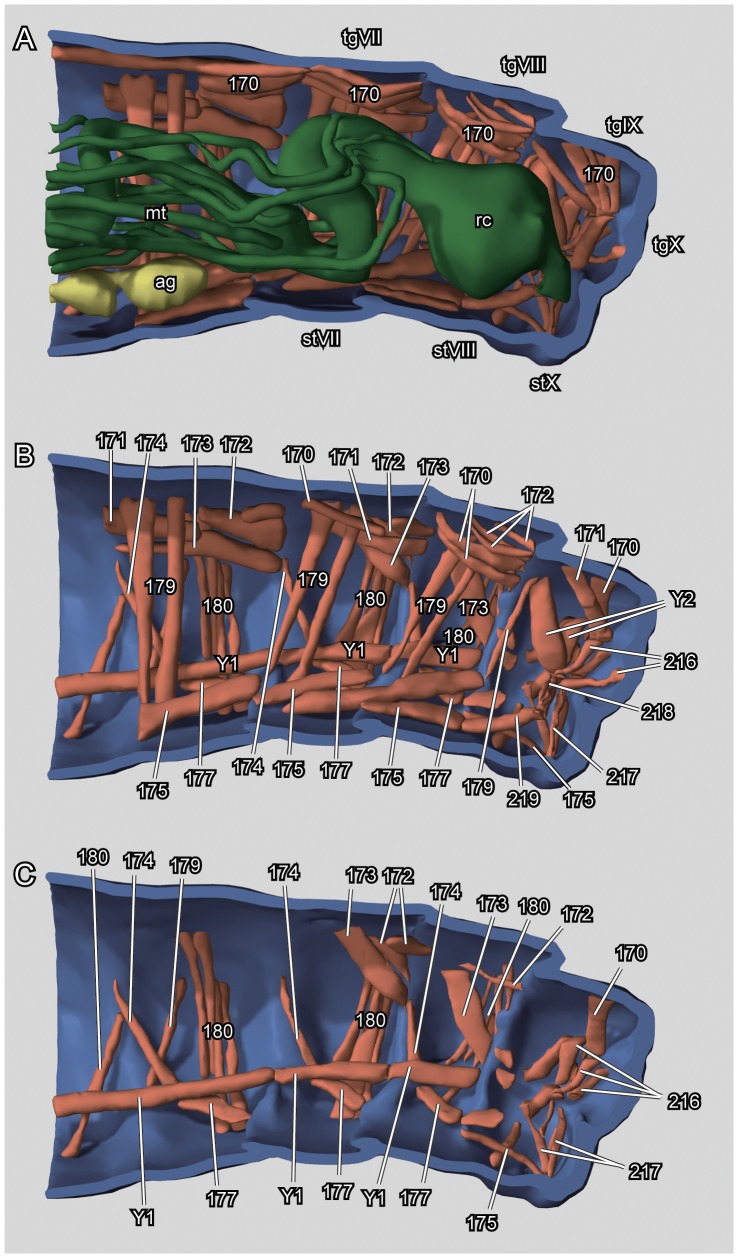
*L. vesicatoria*, abdomen, midsagital cut, mesal view, 3D reconstruction. Abbreviations: ag: abdominal ganglion; mt: Malphigian tubules; rc: rectum; stVII–X: sternite VII–X; tgVII–X: tergite VII to X; abdominal musculature in Arabic numbers and Y1 and Y2.

Body length 2.2–2.5 mm. Colour of head (Figs. 1, 2, 3, 4, 5, 6, 7, 8), legs and other sclerotized body parts light brown; colour of membranous areas yellowish. Tergites of thorax (Figs. 1, 3, 8, 9) and abdomen (Figs. 1, 10, 11) distinctly developed, well sclerotized, not divided into separate sclerites (Fig. 9); thoracic tergites subdivided medially by zone of weakness functioning as ecdysial suture. Sternites membranous, with the exception of abdominal sternite IX and small areas around sternal setae (Figs. 3, 10). Cuticle reticulate with transverse polygonal meshes, more evident around tergites. Conspicuous spiracles present on mesothorax and abdominal segments I–VIII (Figs. 9, 10). Setation well developed; pattern as shown in Figs. 1, 2, 3, 9 and 10.

#### 2.1 Head capsule

Not or slightly retracted into prothorax (Figs. 1, 2), depending on contraction of cervical muscles; posterior region fully exposed in moderately declined resting position of head. Prognathous, well sclerotized, moderately flattened, slightly rounded laterally, evenly narrowing towards foramen occipital (Fig. 2). Dorsal surface distinctly convex, broader than long, distinctly longer than slightly concave ventral side of head capsule. Frontal suture distinct, V-shaped posteriorly (Fig. 2), anteriorly almost perpendicularly extending towards external margin of head at level of antennal bases. Coronal suture long. Dorsal median endocarina absent. Frontoclypeal transverse strengthening ridge (frontoclypeal suture) not developed. Anterior margin of clypeal region almost subtriangular, slightly convex. One pair of relatively large stemmata present on dorsolateral head region, separated from posterior margin of antennal base by about twice the diameter of the moderately convex lens (Fig. 2). Antennal insertion area placed at anterolateral edge of head capsule, about four times as large as stemmata; margin forming a slightly developed bulge dorsally and laterally; on ventral side separated from adjacent parts of head capsule by a semi-circular concavity (Fig. 2). Maxillary grooves almost absent, extremely shallow. Gula fused with submentum anteriorly; widening posteriorly; anteriorly separated from genal regions by low internal ridges (not recognisable externally on SEM micrographs), which are obliterated posteriorly; thus lateral and ventral regions of head capsule apparently forming a rigid, undivided structural unit (Fig. 5). Hypostomal rods and ventral epicranial ridge absent. Posterior tentorial grooves recognisable as indistinct, longitudinal fissure-shaped structures in central region of ventral head capsule, forming landmark between fused gular and submental regions (Fig. 5). Distinct transverse ridge separating narrowed neck region from main part of head capsule absent. Narrow, inconspicuous semi-circular bulge encloses fairly wide and oval foramen occipitale dorsally and dorsolaterally; medially interrupted by posterior end of coronal suture. Vestiture of setae well developed; pattern as shown in Figs. 2 and 4. Most setae very long and thin. Five pairs of setae form a transverse row posterad of clypeal region; four pairs of setae form curved row along mesal side of frontal suture; one pair of seta inserted below posterior part of frontal suture and laterad of coronal suture; five pairs of setae form one longitudinal row along coronal suture, the anterior one longest, the other four very short; five setae arranged along each stemma dorsally; six setae insert on each side of lateral cephalic region, forming two curved longitudinal rows, almost parallel to each other.

#### 2.2 Endoskeleton

Anteriorly converging low gular ridges continuous with narrow postoccipital ridge posteriorly; anteriorly continuous with posterior tentorial arms. Posterior arms strongly converging anteriorly. Dorsal arms present, very flat and unsclerotised, mesally connected with epipharynx, dorsally connected with head capsule by fibrillar structures. Anterior arms and tentorial bridge absent.

#### 2.3 Labrum

Well-developed but partially fused to clypeus in middle region. Almost vertically oriented, partly overlapping with dorsal mandibular surface. Shape transverse, but distinctly narrower than clypeal region; anteriorly rounded. With vestiture of long setae on dorsal surface and close to anterior margin (Fig. 4); three parallel rows of setae inserted from base to anterior margin; base and middle row with four pairs of setae each; distal part with three pairs of setae.

Musculature (muscles not listed are absent) (Fig. 8): 0lb5 (M.7): M. labroepipharyngalis, absent; 0lb2 (M.9): M. frontoepipharyngalis, strongly developed, O ( = origin): laterally on frontal region, I ( = insertion): strongly developed lateral apodemes (tormae) arise from ventral section of epipharynx.

#### 2.4 Antenna

Short, 3-segmented. Antennomere 1 slightly broader than 2 and similar in length, about half as long as 3. Antennomere 2 more than twice the width of 3, asymmetrical, longer along dorsal margin; apex oblique with two long and two short setae. Antennomere 3 elongate, narrow, cylindrical. Apical seta whip-like, slightly shorter than entire head capsule; four additional subapical setae shorter than antennomere 3. Sensory appendix of antennomere 2 large, conical, inserted on short ventral half of segment; less than half as long as apical antennomere (Fig. 4).

Musculature (Fig. 8): 0an1 (M.1): M. tentorioscapalis anterior, well developed, O: dorsal wall of head capsule, adjacent to fibrillar attachment structures of dorsal tentorial arm; I: ventrally on base of scapus; 0an2-4 (Mm. 2–4): M. tentorioscapalis posterior/lateralis/medialis, one well developed bundle, O: dorsal wall of head capsule, laterad 0an1, I: posteriorly on base of scapus.

A thin, oblique muscle associated with an antennal circulatory organ originates at the fibrillar attachment structures of the dorsal tentorial arm.

#### 2.5 Mandibles

Symmetrical. Robust proximal part approximately triangular in cross section, trapezoid in lateral view. Mandibular bases widely separated from each other. Distal part long and slender, falcate, with single acuminate apical tooth (Fig. 6); widely overlapping in resting position; moving in horizontal plane. Ventral mandibular surface concave. Mola, retinaculum or prostheca absent. Mesal margin weakly serrate. Two moderately long setae inserted on external surface. Two pits present meso-dorsally.

Musculature (Fig. 8): 0md1 (M.11): M. craniomandibularis internus, largest muscle of head, O: dorsally and postero-dorsally from head capsule, I: adductor tendon; 0md3 (M.12): M. craniomandibularis externus, large muscle, O: laterally from head capsule, I: abductor tendon.

#### 2.6 Maxillae

Articulating area almost completely reduced, extremely shallow. Cardo vestigial, apparently represented by narrow, largely membranous bulge between basal stipital margin stipes and margin of reduced maxillary groove; with one short seta on small, slightly more sclerotized area. Pad-like element of mesal articulating area absent. Stipes comparatively large, diverging towards base, roughly trapezoid, with very shallow baso-lateral concavity; with two long and two short setae ventrally, and ca. nine setae mesally; articulating area of palp oblique, antero-laterally directed (Fig. 5). Mala simple, small, greatly reduced. Palpi short and stout, 3-segmented, ventrally directed; palpomeres 1 and 2 very short and wide, almost cup-shaped; palpomere 2 with a long seta inserted laterally; palpomere 3 enlarged, base with a short seta mesally; widening distally, flattened, with flattened dorsal apical region; surface of apical region with long and medium sized setae at margin and densely set with sensorial papillae; dorsal surface with reticulate pattern (Fig. 5).

Musculature (Fig. 8): 0mx2 (M.19): M. craniolacinialis, O: ventral wall of head capsule posterad of mesal subcomponent of M. 11, I: dorsolaterally on the base of the lacinia; 0mx3 (M.17): M. tentoriocardinalis, two strongly developed parallel bundles, O: posterior tentorial arm, I: ventral surface of cardo and stipes and mesal stipital edge; 0mx8/9/10 (Mm 22/23): M. stipitopalpalis externus/internus, O: ventrally on stipital base, I: ventrally and dorsally on base of palpomere 1.

#### 2.7 Labium

Submentum posteriorly fused with gula (see above), with one pair of setae; partly enclosed by low lateral ridge, but largely fused with adjacent parts of head capsule (lateral border not recognisable on SEM micrographs). Mentum small, trapezoid, slightly diverging anteriorly; with one pair of short setae; membrane connecting mentum and prementum laterally exposed, with minute spine-like surface modifications. Prementum very small, narrowing towards base. Palpi 2-segmented, ventrally directed; outer margin of first segment about 2.5 times as long as inner margin; palpomere 2 longer than broad, twice as long as 1; inner margin slightly longer than outer margin, parallel-sided, evenly rounded at apex; apical region with ca. 12 regularly arranged small spine-like sensilla, two small, characteristic dorso-mesal papilla, and a central larger 2 segmented sensillum (Fig. 5).

Musculature (Fig. 8): 0la5 (M.29): M. tentoriopraementalis inferior, one moderately large, almost vertical bundle, O: apical part of posterior tentorial arm, I: ventro-mesally on hind margin of prementum; 0la8 (M.28): M. submentopraementalis, O: medially from posterior postmental margin, I: ventrolaterally on premental base.

#### 2.8 Epipharynx and hypopharynx

Anterior epipharynx slightly convex, with shallow median edge and strong paramedian sclerotized longitudinal rods. Intermediate part forming very short preoral chamber together with mandibular bases and intermediate region of hypopharynx; shape of chamber subtrapezoid in cross section. Anterior part of hypopharynx adjacent with dorsal side of prementum; posterior hypopharynx fused with epipharynx laterally, thus forming the floor of a prepharyngeal tube; prepharynx shaped like a flattened U in cross section, with very distinct upper edges.

Musculature (Fig. 8): 0hy1 (M.41): M. frontohypopharyngalis, well developed, O: central area of frons, I: postero-laterally on hypopharynx by means of a strong tendon; 0ci1 (M.43): M. clypeopalatalis, cibarial dilator composed of thin bundles, O: successively on the posterior clypeal region, I: successively on epipharynx, close to median line. Well-developed transverse muscles separate the bundles of 0ci1.

#### 2.9 Pharynx

Fairly wide, almost quadrangular in cross section. Dorsolateral, lateral and ventrolateral folds of posterior pharynx indistinct, more distinct towards anatomical mouth. Anterior pharynx continuous with prepharyngeal tube.

Musculature (Fig. 8): 0bu2 (M.45): M. frontobuccalis anterior, well-developed bundle, O: central region of frons; I: dorsad the anatomical mouth, adjacent to insertion of 0hy1; 0bu3 (M.46): M. frontobuccalis posterior, thin transversely arranged parallel bundles, O: frons, between origins of 0lb2 and 0bu2, I: dorsally on precerebral pharynx, close to anatomical mouth. Well-developed ring muscles present over entire length of pharynx. 0ph1 (M. 51): M. verticopharyngalis: absent; 0ph2 (M. 52): M. tentoriopharyngalis, absent.

#### 2.10 Cerebrum and suboesophgeal ganglion (Figs. 7, 12)

Cerebrum elongate, partly shifted to postcephalic body, slightly asymmetric, posteriorly reaching anterior part of prothorax. Suboesophageal ganglion located in prothorax, not fused with prothoracic ganglion.

#### 2.11 Thorax (Figs. 1, 3, 4, 8, 9)

Tergites well sclerotized. Sternal and pleural regions largely membranous or semimembranous, without distinct sclerites. Furcae present in all three segments. Most setae minute, few of medium length; ventral side largely devoid of setae. Medium zone of weakness (ecdysial suture) complete on pronotum, almost complete on mesonotum, distinctly shortened on metanotum. Legs similar on all three segments, slender, tapering distally, 5-segmented; femur not enlarged in its middle region, shorter than tibia; fore femur slightly longer than meso- and metafemora; femoral setae normally shaped; fore- and mesofemoral seta 1 twice as long as seta 2; tibiae tapering towards apex; apical setae of metatibiae slightly longer than width at tibial apex; tarsungulus slender, evenly tapering towards apex, with two dense and regular rows of short setae; apex with claw and two very slightly curved setae of medium length forming a trident.

#### 2.12 Prothorax (Figs. 1, 3, 8, 9)

Segment broader than head. Anteriorly with broad membranous collar, with fairly indistinct dorsolateral fold and distinct ventral fold, slightly more distant from anterior margin. Pronotum transverse, 1.5 as long as meso- and metanotum; posteriorly with distinct transverse bead; Posterior dorsal part of prothorax membranous; cuticle unpigmented, moderately sclerotized, but distinctly delimited anteriorly, posteriorly and laterally. Discrete pleural or sternal sclerites absent. Pleurocoxal joint distinct, with almost horizontal, slightly curved line present above it. Pleural suture recognisable but very short. Profurca very small, located at the postero-mesal coxal rim. Legs well developed, distally with elongated, trumpet-shaped empodium. Prothoracic ganglion present as a discrete separate structure (see above).

Musculature (Fig. 8): The muscles of the thorax are assigned with the token m and continuous numbers (1–). A homologisation with the thoracic musculature with other (adult) insects is problematic and a muscular nomenclature for holometabolan larvae is not available.

Dorsal longitudinal muscles: m1, strong, O: dorso-mesal rim of postocciput, I: dorsal region of prophragma. m2, O: postocciput, directly mesad m1, I: ventro-lateral region of prophragma. m3, bipartitioned, O: postocciput, ventro-laterad m1, laterad m1, I (m1a): posteriormost region of pronotum, I (m1b): middle region of pronotum, anterad m1a, F: depressor and retractor of prothorax. m4, O: dorso-mesally in the middle region of the pronotum, I: prophragma, directly laterad m1. m5, O: posterior third of pronotum, posterad m4, I: ventral part of prophragma. m6, O: posterior margin of pronotum, I: prophragma, directly dorsad m4.

Ventral longitudinal muscles: m7, thick bundle, narrowing towards attachment area, O: ventro-lateral margin of postocciput, I: ventro-laterally on segmental border, between pro- and mesothorax. m8, O: ventro-mesally on postocciput, I: furca. m9, O: antero-ventral sternal region, postero-laterad m8, I: furca, directly laterad m8.

Dorsoventral muscles: m10, O: posterior half of pronotum, I: ventro-laterally on posterior margin of head capsule. m11, O: lateral prophragma, I: posterior head capsule, slightly postero-mesad the insertion of m10. m12, O: anterior third of pronotum, I: postero-ventral margin of head capsule, directly laterad m10. m13, O: lateral postoccipital margin, I: antero-lateral sternal region. m14, O: anterior postoccipital margin, directly dorsad m13, I: middle region of sternite, between anterior margins of coxae. m15, O: dorsolateral body wall, slightly anterior to prophragma, I: slightly laterad the insertion of m7. m16, O: ventral prophragma, I: profurca.

Tergopleural muscles: m17, a stout muscle, O: anterior pronotum, I: lateral body wall. m18, O: posterior pronotum, I: lateral body wall, slightly posterad m27.

Pleural muscles: m19, a muscle with two heads, O: with two heads on lateral body wall, first head anterad the second, I: ventro-laterally on segmental border between pro- and mesothorax, slightly anterad m7. m20, extends along ventro-lateral body wall.

Tergocoxal muscles: m21, strong muscle with two heads, O: pronotum, I: posterior coxal rim. m22, broad muscle, O: with broad origin on pronotum anterad and posterad m25, I: slightly laterad m25. m23, O: pronotum, I: slightly anterad m22. m24, slender muscle, O: pronotum, I: anterior coxal rim.

Pleurocoxal muscles: m25, O: latero-ventral body wall, ventrad m19, I: posterior-mesal coxal rim, mesad m19. m26, short and stout muscle, O: latero-ventral body wall, directly posterad m20, I: posterior-lateral coxal rim, laterad m19. m27, long and slender muscle, O: lateral body wall, I: mesal coxal rim. m28, O: directly anterad m22, I: anterio-mesal coxal rim, slightly anterad m22.

#### 2.13 Mesothorax (Figs. 1, 3, 8, 9)

General structure and legs similar to prothorax. Mesonotum shorter than pronotum, with one pair of spiracles in anterior half. Dorsal side with three parallel rows of medium-sized setae from anterior to posterior regions; first row with two pairs of setae, second with four pairs, third with three pairs; ventral side with two pairs of setae along midline from anterior to posterior margin. Dorsal side of posteriormost segmental region membranous. Pleural and sternal sclerites indistinct. Small furca present postero-mesad the coxa.

Musculature (Fig. 8): Dorsal longitudinal muscles: m29, with several bundles, O: prophragma, I: mesophragma. m30, O: mesophragma, I: middle region of mesonotal area.

Ventral longitudinal muscles: m31, long and slender muscle, O: profurca, I: mesofurca. m32, long and slender, O: ventral segmental border between pro- and mesothorax, posterad m7, I: mesofurca. m33, long and slender, O: ventral segmental border between pro- and mesothorax, posterad m7, I: ventral segmental border between meso- and metathorax. m34, muscle with two heads, O: mesofurca, I: ventral segmental border between meso- and metathorax. m35, O: mesofurca, I: ventral mesophragma.

Dorsoventral muscles: m36, O: antero-lateral mesonotum, I: ventral segmental border between pro- and mesothorax, close to origin of m32. m37, stout muscle, O: posterior mesonotum, I: ventral body wall at segmental border between meso- and metathorax. m38, O: mesonotum, I: ventral body wall, anterad the coxa.

Tergocoxal muscles: m39, O: mesonotum, I: postero-mesal coxal rim. m40, O: mesonotum, I: anterio-mesal coxal rim. m41, O: mesonotum, anterad m44, I: postero-lateral coxal rim.

Tergopleural muscles: m42, O: anterior mesonotum I: lateral pleural region. m43, O: pronotum, posterad m42, I: lateral pleural region. m44, O: posterior pronotum, I: lateral pleural region, slightly ventral m43. m45, O: mesonotum, close to mesophragma I: lateral body wall.

Pleurosternal muscles: m46, O: pleural region, I: ventral segmental border between pro- and mesothorax.

Pleurocoxal muscles: m47, O: pleural region, I: antero-lateral coxal rim. m48, O: pleural region, I: postero-lateral coxal rim. m49, O: pleural region, I: trochanter.

Sternocoxal muscles: m50, O: mesofurca, I: mesal coxal rim. m51, O: ventral segmental border between pro- and mesothorax, directly laterad m45, posterior coxal rim.

#### 2.14 Metathorax (Figs. 1, 3, 8, 9)

General structure, legs and chaetotaxy similar to mesothorax. Metanotum reaches further ventrad than mesonotum. No spiracle present. Membranous area present between metanotum and abdominal tergite I. Small furca present.

Musculature (Fig. 8): Dorsal longitudinal muscles: m52, two bundles, O: mesophragma, I: metaphragma. m53, two distinct bundles, O: metaphragma, I: metanotum.

Ventral longitudinal muscles: m54, long and slender muscle, two bundles, O: mesofurca, I: metafurca. m55, O: ventral segmental border between meso- and metathorax, posterad m35, I: metafurca. m56, O: mesofurca, I: ventral segmental border between metathorax and abdominal segment I. m57, O: metafurca, I: ventral segmental border between metathorax and abdominal segment I.

Dorsoventral muscles: m58, O: antero-lateral metanotum, I: ventral segmental border between meso- and metathorax, close to the insertion of m34. m59, O: posterior metanotum, I: ventral body wall at segmental border between metathorax and abdominal segment I. m60, O: metafurca, I: ventral metaphragma.

Tergocoxal muscles: m61, O: lateral metanotum, I: anterior coxal rim.

Tergopleural muscles: m62, O: antero-lateral region of metanotum, slightly laterad m58 I: lateral pleural wall. m63, O: metanotum near metaphragma, I: lateral body wall.

Pleurosternal muscles: m64, O: lateral pleural wall, I: ventral body wall, antero-mesad the coxa. m65, O: lateral-ventral body wall, I: sternal region anterad the coxa.

Pleurocoxal muscles: m66, O; lateral body wall, I: mesal coxal rim, close to m59. m67, O: lateral body wall, directly dorsad m61, I: anterio-mesal coxal rim, anterad m70. m68, O: latero-ventral body wall, I: lateral coxal rim. m69, O: latero-ventral body wall, directly posterad m68, I: trochanter.

Sternocoxal muscles: m70, O: metafurca, I: posterior coxal rim. m71, O: metafurca, I: mesal coxal rim.

#### 2.15 Abdomen

With 9 segments of more or less uniform length and a strongly reduced tenth segment. Terga well sclerotized, with antero-median group of minute setae and row of setae along posterior margin. Pleurites indistinct. Urogomphi absent. Apex of abdomen with pair of long caudal setae (Fig. 10). Eight abdominal ganglia present, ganglia VII and VIII closely adjacent but not fused.

#### 2.16 Segments I–VIII

Segments similar in shape, cylindrical, shorter than thoracic segments (Fig. 1). Cuticle pale brown, moderately sclerotized. Sclerites distinctly delimited anteriorly, posteriorly and laterally. Abdominal spiracle I smaller than mesothoracic spiracle; abdominal spiracles I–VI subequal in diameter, VII and VIII slightly smaller; spiracle VIII much smaller than spiracle I. Tergites with three pairs of medium-sized setae along median region of posterior margin; two additional pairs of long setae inserted postero-laterally; one pair of setae inserted on pleurite. Sternite with one pair of medium-sized setae on central region, and close to them one additional pair of minute setae; two pairs of longer setae inserted on posterior margin (Fig. 10).

Musculature (Fig. 11): M. 170 (M. antecosta-antecostalis uronotum medialis): O: anterior region of tergite; I: anterior margin of following tergite; F: retractor of the following segment. M. 171 (M. antecosta-antecostalis uronotum lateralis): M. antecosta-antecostalis uronotum lateralis. O: anterior region of tergite, close to lateral margin; I: anterior margin of following tergite, close to lateral margin; F: lateral retractor of following segment. M. 172 (M. uronoto-antecostalis obliquomedialis): O: posterior part of tergite; I: anterior edge of following tergite; F: transverse rotation of abdominal segments. M.173 (M. uronotoantecostalis obliquolateralis): O: posterior part of tergite; I: anterior edge of following tergite, ventro-laterad to M. 172; F: transverse rotation of abdominal segments, together with M. 173. M. 174 (M. uronotoantecostalis): O: ventral tergite; I: latero-ventral region, close to insertion of M. 177. M. 175 (M. antecosta-antecostalis urosterni medialis): O: ridge on sternite; I: anterior edge of following sternum; F: retractor of sterna. M. 177 (M. antecostaantecostalis urosterni lateralis): O: sternite ridge; I: anterior edge of following sternum, laterad to M. 175; F: retractor of sterna, together with M. 175. M. 179 (M. urotergosternalis interus primus), several bundles: O: tergite, beneath M. 170; I: base of lateral sternal apodeme; F: compressor of abdomen. M. 180 (M. urotergosternalis interus secundus), in several bundles: O: posterior tergum; I: posterior sternal region. M. 216 (M. tergorectalis dorsalis): O: middle of tergite X; I: latero-dorsal rectum; F: dorsal dilator of the rectum. M. 217 (M. tergorectalis lateralis), in several bundles: O: latero-ventral region of segment X; I: ventro-lateral rectal wall. F: dilator of rectum. M. 218 (M. epiproctoanalis), in several bundles: O: on tergite X; I: dorsal anal wall; F: dilator of the anus. M. 219 (M. paraproctoanalis): O: sternite IX; I: latero-ventrad of anus; F: lateral dilator of the anus. Y1: ventral longitudinal intersegmental muscles not described by v. Kéler (21). Y2: rectal muscle not described by v. Kéler (21): O: tergite IX; I: lateral rectal wall.

#### 2.17 Segments IX–X

Segment IX well developed, slightly narrower than VIII. Segment X distinctly reduced, slightly conical (Fig. 10), with protruding cuticular structure with unclear homology (referred to as pygopod in the following; [22:] anal vesicle; [16]: pygopod).

#### 2.18 Postcephalic gut (Figs. 11, 12)

Oesophagus short, almost round in cross-section, with thin intima and ring muscle layer. Midgut straight and relatively wide; wall formed by cylindrical cells in anterior part and cubic cells in posterior part. Hindgut relatively short, forming a loop; round in cross-section, with ring muscles. Rectum short, wider than hindgut; wall much thicker.

#### 2.19 Malpighian tubules (Fig. 11)

Four Malpighian tubules enter the digestive tract at the anterior end of the hind-gut, extending through large areas of postcephalic body cavity, forming numerous short coils along hind gut; distal part entering below connective tissue covering posterior hind gut, ending at rectal constriction.

#### 2.20 Circulatory system and fat body

Tubular heart present in abdominal segments. Aorta extending through thorax, terminating at postcephalic region.

Fat body lobes fill out extensive parts of body cavity, mainly accumulated in abdomen. Fat body cells variously shaped, with large and small inclusions.

#### 2.21 Tracheal system

Longitudinal tracheal trunks developed in thoracic and abdominal segments.

## Discussion

The main focus of this study is the detailed documentation of external and especially internal features of the primary larva of *Lytta vesicatoria*. Consequently the characters observed will be discussed in a morphology based sequence, with respect to their phylogenetic implications, the larval groundplan of Meloidea, possible correlations to phoretic or parasitoid habits, and possible effects of miniaturisation. External features of first instar larvae where already treated in a considerable number of detailed studies (e.g., [4]; [23]; [24]; [25]; [26]; [27]; [28], [29]; ) and also in an extensive work on the intrafamiliar relationships and the evolution of phoresy [5; see also 2]. Therefore, the systematic placement of *Lytta* and the phylogeny within Meloidae will be only marginally covered in the following.

A 1^st^ instar larva of the triungulin type is almost generally found in meloid larvae, with the notable exception of *Eletica* and *Iselma* [5]. This condition, linked with a specialisation on acridoid eggs or larvae and provisions of bees [5]; [26], is likely an autapomorphy of Meloidae excl. Eleticinae. The campodeid body shape of *Lytta* and Meloinae in general (e.g. [5]; [34]) is apparently a groundplan feature of Meloidae. The derived counterparts are found in larvae of the specialised Nemognathinae, which are generally phoretic (excl. *Stenodera* which was removed from this group; [5]). Larvae of this subfamily have a distinctly navicular body, somewhat similar to the condition found in first instar larvae of Strepsiptera and the tenebrionoid family Rhipiphoridae. Larvae of these two groups are heavily sclerotized, whereas a moderate degree of sclerotization with large exposed membranous areas is apparently a groundplan feature of Meloidae. The larval body shape of Eleticinae is similar to conditions occurring in other tenebrionoid lineages according to Bologna & Pinto [5]. Nevertheless, it is conceivable that the orthosomatic body with the greatest width at the middle region of the abdomen [33] is a derived condition and a possible autapomorphy of the subfamily.

The setation is well developed in *Lytta* and in the groundplan of Meloidae [5]; [26], compared to the condition in Rhipiphoridae. However, apparently it tends towards reduction, especially in the phoretic Nemognathinae ([26]: fig. 34.705).

The head capsule of first instars of *Lytta* displays only one feature typical and possibly autapomorphic for Tenebrionoidea, the anterior shift of the posterior tentorial arms [11]. A posteriorly diverging gula (fused with the submentum) is another possible autapomorphy of Tenebrionoidea [11]. However, the lateral margin of this sclerotized region is hardly recognisable in *Lytta* (Fig. 5). A gula with parallel sides or even slightly narrowing towards the foramen occipitale occurs in primary larvae of the presumably basal Eleticinae [5]; [35] and also in other meloid taxa (e.g., [36]): *Meloe*; [27]: *Austrolytta*). It is conceivable that this is a derived groundplan feature of the family.

The head capsule of *Lytta* is largely characterised by plesiomorphic features. Its moderate length and its moderately rounded lateral sides are very likely plesiomorphic. A head narrower than the thorax is arguably a derived feature of Eleticinae [33] and the characteristic helmet-shaped, elongated and posteriorly widening head capsule of larvae of Nemognathinae ([26]: fig. 34.705; [32]) is apparently autapomorphic for this subfamily. The head of the phoretic larvae of Nemognathinae is superficially similar to that of first instar larvae of Rhipiphoridae, which is also elongated, anteriorly rounded, and widening towards its posterior margin. It is apparent that this structural affinity has evolved independently in both families.

Other plesiomorphic character states of *Lytta* are the presence of a long coronal suture and distinctly developed frontal sutures, and the absence of a median endocarina. The endocarina is apparently an autapomorphy of Tetraonycinae within Meloidae [5], but has evolved several times independently within Tenebrionoidae (e.g., Anthicidae). The absence of a transverse ridge on the vertex of *Lytta* and many other meloid larvae is plesiomorphic, and the corresponding apomorphic condition is linked with phoretic habits [37]. The frontal coronal and frontal sutures are clearly present in *Lytta* and other larvae of Meloinae (e.g., [34]), but varying degrees of reduction occur, especially in Nemognathinae ([26]: fig. 34.705; [37]). They are very indistinct or absent in Eleticinae [33], another derived feature of this subfamily. The epicranial sutures are also absent in the parasitic primary larvae of Strepsiptera and Rhipiphoridae. This may be related to miniaturisation and an increased mechanical stability, at least in strepsipteran larvae, which are extremely reduced in size [38], lack a tentorium, and use the head capsule to penetrate the host’s body wall. The absence of hypostomal rods and ventral epicranial ridges is occurring in different lineages of Tenebrionoidea (e.g., [11]). The shortening of the maxillary groove is also a derived condition occurring in different tenebrionoid families (e.g., [11]). It is almost completely absent in *Lytta* (Fig. 5), much less distinct as for instance in *Meloe* ([26]: fig. 34.699). The presence of a large U-shaped ventromedian emargination in primary larvae of *Eletica* [33] is possibly an autapomorphy of Eleticinae. This feature is obviously related with the formation of a maxillolabial complex resembling that of elateriform or cleroid larvae [10]; [39], another possible autapomorphy of Eleticinae.

A derived feature of *Lytta* and probably a groundplan feature of Meloidae is the reduced condition of the tentorium, i.e. the loss of the anterior arms and the bridge, and the reduced condition of the dorsal arms. A more or less far-reaching reduction of the head capsule is common in larvae of Tenebrionoidea [11]. The thin and membranous dorsal arm is arguably a groundplan feature of this lineage, whereas the loss of the anterior arms and bridge probably occurred several times independently (e.g., Melandryidae, Ulodidae [11]). An unusual derived feature is the connection between the dorsal arm and the epipharynx. The distribution of this apomorphic character state is unclear at present.

The presence of a maximum number of two pairs of stemmata [26] is arguably a groundplan apomorphy of Meloidae. This is in contrast to first instar larvae of Rhipiphoridae, which possess four or five pairs of stemmata. Two stemmata occur in larvae of the subfamily Nemognathinae [5] and intuitively one would assume that this is a plesiomorphic condition. However, it is conceivable that the number was secondarily increased in the groundplan of this lineage. The complete loss of eyes is apparently another autapomorphy of Eleticinae [5]; [33]. The dorsal position of the stemmata is likely a groundplan autapomorphy of the entire family, with reversal in phoretic groups as pointed out by Bologna & Pinto [5].

A derived feature of larvae of *Lytta* and all other meloids is the absence of the frontoclypeal strengthening ridge (e.g., [26]; [33]; [37]), but this condition is found in most groups of Tenebrionoidea [11]. A feature of *Lytta* slightly differing from the presumptive groundplan of Meloidae is the partial fusion of the labrum with the anterior frontoclypeal margin. The labrum is entirely free in Eleticinae [33] and some other members of the group (e.g., *Austrolytta*; [40]). The loss of the intrinsic labral muscle in *Lytta* (0lb5) is also apomorphic, but this condition occurs in many groups of beetles (e.g., [11]). The presence of a well-developed pair of external retractors (0lb2) is obviously a plesiomorphic feature. The complete fusion and immobilisation of the labrum, likely linked with the loss of extrinsic muscle, is possibly an autapomorphy of Nemognathinae [26]; [37]. The labrum is generally fused with the head capsule in 1^st^ instar larvae of Rhipiphoridae [26] and also in Strepsiptera, where labral muscles are completely missing [38].

A short 3-segmented antenna as it is found in *Lytta* is likely close to the groundplan of Meloidae. The presence of an elongated terminal seta is arguably a groundplan apomorphy of the family. However, short terminal setae occur in different groups such as for instance *Cyaneolytta* or *Pseudomeloe* [5], arguably a result of reversal. The presence of elongate terminal setae in Rhipiphoridae is apparently a result of parallel evolution. In which way this apomorphic condition is linked with specific functions in both groups is unclear. The subconical sensory appendage of the penultimate antennal segment of *Lytta* (Figs. 2, 4; 5: fig. 13F) appears unusually broad compared to the condition in most other meloid larvae (e.g., [27]: fig. 7; [32]: fig. 9), but is very similar to the sensory appendage of *Alosimus* ([34]: 2E, F). A moderately sized and relatively slender appendage is likely present in the groundplan of the family. A greatly enlarged, curved and saccular sensillum is likely an autapomorphy of the genus *Eletica* ([5]: absent in *Iselma*; [33]). The presence of only two extrinsic antennal muscles is a derived feature. Three antennal muscles are present in other tenebrionoid families, even though the antennae may be even shorter than in meloid larvae (e.g., Melandryidae, *Orchesia*; [11]). The interpretation of the presence of a muscle of the antennal circulatory organ is difficult at the present state of knowledge. This muscle has not been described in any studies on the cephalic anatomy of beetle larvae so far (e.g., [8]; [11]).

The mandibles of *Lytta* display a combination of plesiomorphic and specialised features. The musculature is similar to conditions found in other tenebrionoid larvae [11]. The horizontal plane of movement is apparently a plesiomorphic feature and the corresponding apomorphic state, i.e. more or less vertically oriented mandibles, a derived feature characterising phoretic meloid larvae. The falcate shape of the distal part of the mandibles and the reduced mola are arguably autapomorphies of Meloidae, even though similar conditions also occur in first instar larvae of Rhipiphoridae [41]. The wide separation at the base is likely an apomorphy of Meloidae excluding Eleticinae [5], and also the secondary absence of a group of microtrichia according to the same authors. However, whether the spine-like setae at the base of the mola of anthicid larvae [42] are really homologous to the microtrichia of eleticine larvae (coding in [5]) appears doubtful. The modifications of the mandibles belonging to the meloid groundplan, especially the falcate shape and the reduction of the mola, are apparently linked with specialised predaceous habits.

The maxillae of *Lytta* and most other meloid larvae have maintained a limited lateral movability. The presence of a maxillolabial complex is likely an autapomorphy of Eleticinae as pointed out above. The absence of a pad-like and sclerotized articulatory area in *Lytta* and other meloid larvae is a plesiomorphic condition. The pad is present in Anthicidae and some related groups (e.g., Pythidae, Boridae, Prostomidae [11]; [42]). The presence of a partly reduced but still recognisable cardo is likely a groundplan feature of Meloidae. The cardo is only very indistinctly separated from the stipes in Eleticinae and its complete absence as a separate maxillary element is likely a synapomorphy of Nemognathinae and Tetraonycinae [5]. The reduced condition of the mala is probably an autapomorphy of Meloidae [5]; [33]. The number of three distinct maxillary palpomeres is a groundplan feature of Meloidae. A reduced number of two is very likely another autapomorphy of Eleticinae [5]; [33], with parallel evolution in some Rhipiphoridae [26]. The strongly shortened palpomeres 1 and 2 of *Lytta* are apparently apomorphic conditions compared to most other meloid larvae (e.g., *Meloe*; [26]: fig. 34.699; *Austrolytta* [27]: figs. 4–6). A somewhat similar condition is present in *Dictyolytta* ([5]: fig. 11) and also in genera assigned to *Lydina* [34]. The highly unusual shape of the broad and spoon-shaped apical palpomere, with a concave dorsal side with scale-like surface modifications, and a very specific arrangement of sensilla on the apical region is a complex apomorphic condition shared with larvae of *Oenas* ([34]: fig. 3F) and other genera assigned to *Lydina* [34]. The maxillary musculature of *Lytta* is moderately simplified. M. craniocardinalis is absent, but this is also the case in different other tenebrionoid lineages (e.g., Colydiidae, Melandryidae, Ulodidae [11]). The loss of intrinsic maxillary muscles (Mm. stipitolacinialis and -galealis) is a common feature in beetle larvae (e.g., [8]; [11]; [43]; [44], [45]; [46]).

**Figure 12:**
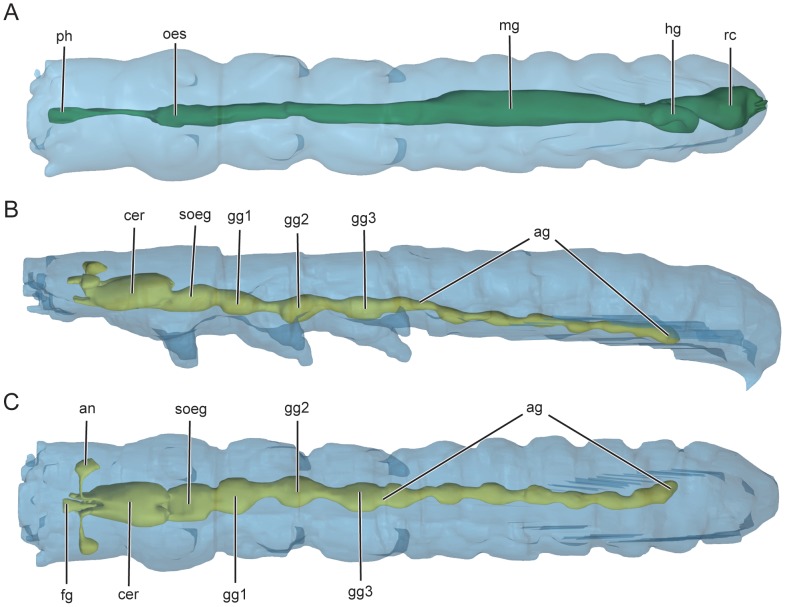
Digestive (A) and nervous (B, C) systems of *L. versicatoria*, 3D reconstructions. (A, C) dorsal view; (B) lateral view. Abbreviations: ag, abdominal ganglia; an: antennal nerve; cer, cerebrum; fg: ganglion frontale; gg1,2,3 pro-, meso- and metathoracic ganglia; hg, hind-gut; mg, mid-gut; oes, oesophagus; ph, pharynx; rc, rectum; soes, suboesophageal ganglion.

The labium of meloid larvae is well developed compared to the greatly reduced condition in Rhipiphoridae [26]. However, the labial structures of *Lytta* are apparently distinctly different from the presumptive meloid groundplan, as it is probably displayed by larvae of *Meloe* [26]. The nearly complete fusion of the submentum with the adjacent parts of the head capsule in *Lytta* and also in *Oenas* ([34]: fig. 3B, D) is certainly an apomorphic feature compared to what is found in most other meloid larvae (e.g., [26]: fig. 34.669; [5]: fig. 12D, F), and also the small size and ventral orientation of the prementum. A small and more or less unsclerotised mentum is possibly a derived groundplan feature of Meloidae. It appears even more reduced in *Lytta* than in other groups of Meloidae, such as for instance *Meloe* or *Eletica* [5]; [26]; [33]. The sensilla on the apical region of palpomere 2 is nearly identical to the situation in the genus *Oenas* ([34]: fig. 3D), with a larger 2-segmented sensillum enclosed by a group of smaller sensilla. The equipment of labial muscles is probably close to the groundplan condition of Tenebrionoidea [11]. The loss of one of three premental retractors and of all intrinsic premental muscles is a condition frequently found in this superfamily [11] and also in other lineages of beetles (e.g., [8]; [44], [45]; [46]). The digestive tract of the cephalic region does not show unusual features. The loss of the postcerebral pharyngeal dilators is a derived condition occurring in many lineages of beetles(e.g., [8], [11]; [43]; [44], [45]; [46]). In the case of *Lytta* this is arguably related to size reduction like in other small beetle larvae (e.g., [44]: Micromalthidae; [16]: Ptiliidae). As a result of miniaturisation the brain appears unusually large in relation to the head size and is at least partly shifted to the prothorax. This leaves no space for postcerebral dilator muscles.

The larval thorax of *Lytta* is also mainly characterized by plesiomorphic features. The presence of an almost complete median ecdysial line is apparently a groundplan feature of the family, even though it is completely absent in the presumably basal Eleticinae [33], which is likely another autapomorphy of this family. Its presence in most groups of Tenebrionoidea and other beetle lineages suggest that secondary reduction occurred several times independently in Meloidae, such as for instance in *Tetraonyx* [37]. Another plesiomorphic feature of *Lytta* and most other groups of meloid subgroups is the presence of well-developed tergites. Again, the apomorphic condition is found in Eleticinae [33], the absence of well-defined dorsal postcephalic sclerites. A similar condition has independently evolved in extremely small larvae such as those of *Mikado* sp. (Ptiliidae) or *Sericoderus lateralis* (Corylophidae) [13]; [14], in these cases very likely a result of miniaturisation.

An apparent autapomorphy of Meloidae is the presence of a conspicuous spiracle on the sclerotized mesotergite. The loss of the metathoracic spiracle is a derived condition shared with larvae of Rhipiphoridae, but independent loss is likely. The slender legs of *Lytta* display a combination of plesiomorphic and derived features. The absence of a swollen femur [37] is a plesiomorphic condition, with the derived counterpart occurring in *Epispasta*, *Lyttomeloe*, some species of *Meloe* and few other representative of the family [37]. Apparently the derived condition has evolved several times independently. Another complex of derived features of the larva of *Lytta* and other meloid taxa is the elongate distally tapering tibia, and the distinctly developed triungulin apex of the legs. These derived features suggest phoretic habits, even though larvae of *Lytta* are non-phoretic. It is cannot be fully excluded that a phoretic behaviour is secondarily lost in *Lytta*, together with some but not all morphological modifications related to it.

The thoracic musculature of the moderately miniaturised larvae of *Lytta* is well developed. It is mainly characterized by the leg-moving coxal musculature as was to be expected in an agile first instar larva. Within Tenebrionoidea, the larval thoracic musculature has only been described for *Tenebrio molitor* [12]. Therefore no phylogenetic interpretations are possible at the moment.

A derived feature of the abdomen is the complete loss of the fixed urogomphi, which are present in many groups of Tenebrionoidea. This is possibly an autapomorphy of Meloidae but the urogomphi are also missing in Rhipiphoridae and several other groups of the superfamily. Another derived feature is the presence of one or two conspicuous caudal bristles. Interestingly very similar bristles are also present in strepsipteran 1^st^ instars. However, in *Lytta* they are not equipped with extrinsic muscles and not part of a specialised jumping apparatus. The caudal bristles are usually present in Meloinae but are missing in the basal Eleticinae and also in Nemognathinae [26]; [33].

As a whole, the features we observed in larvae of *Lytta* are largely plesiomorphic, i.e. conform to a presumptive groundplan of Tenebrionoidea. The primary larvae are only moderately reduced in size (e.g., compared to 1^st^ instars of Strepsiptera) and only very few features, such as for instance the posterior shift of brain, can be ascribed to size reduction.

## References

[1] Pinto JD, Bologna MA (1999) The New World genera of Meloidae (Coleoptera): a key and synopsis. Journal of Natural History 33: 569–620.

[2] Bologna MA, Turco F, Pinto JD (2010) 11.19. Meloidae Gyllenhal 1810. In: Leschen RAB, Beutel RG, Lawrence JF, editors. Coleoptera, Beetles, Volume 2: Morphology and Systematics (Elateroidea, Bostrichiformia, Cucujiformia partim). In: Kristensen NP, Beutel RG, editors. Arthropoda: Insecta. In: Handbook of Zoology. Berlin/New York: De Gruyter. Pp. 681–693.

[3] Tagwireyi D, Ball DE, Loga PJ, Moyo S (2000) Cantharidin poisoning due to “blister beetle” ingestion. Toxicon 38: 1865–1869. 10858524 10.1016/s0041-0101(00)00093-3

[4] Selander RB (1960) Bionomics, systematics, and phylogeny of *Lytta*, a genus of blister beetles (Coleoptera, Meloidae). Illinois Biological Monographs 28: 1–295.

[5] Bologna MA, Pinto JD (2001) Phylogenetic studies of Meloidae (Coleoptera), with emphasis on the evolution of phoresy. Systematic Entomology 26: 33–72.

[6] Crowson RA (1981) The biology of the Coleoptera. London, New York etc: Academic Press. 802 p.

[7] Kinzelbach RK (1971) Morphologische Befunde an Fächerflüglern und ihre phylogenetische Bedeutung (Insecta: Strepsiptera). Zoologica 41: 1–128.

[8] Beutel RG (1993) Phylogenetic analysis of Adephaga (Coleoptera) based on characters of the larval head. Systematic Entomology 18: 127–147.

[9] Beutel RG (1994) Phylogenetic analysis of Hydrophiloidea (Coleoptera: Polyphaga: Staphyliniformia) based on characters of the head of adults and larvae. Koleopterologische Rundschau 64: 103–131.

[10] Beutel RG, Pollock DA (2000) Larval head morphology of *Phycosecis litoralis* (Pascoe) (Coleoptera, Phycosecidae) with phylogenetic implications. Invertebrate Taxonomy 14: 825–835.

[11] Beutel RG, Friedrich F (2005) Comparative study of larvae of Tenebrionoidea (Cucujiformia, Coleoptera). European Journal of Entomology 102: 241–264.

[12] Jösting EA (1942) Die Innervierung des Skelettmuskelsystems des Mehlwurms (*Tenebrio molitor* L., Larve). Zoologische Jahrbücher/Abteilung Anatomie und Ontogenie der Tiere 67: 381–460.

[13] Polilov AA, Beutel RG (2009) Miniaturization effects in developmental stages of *Mikado* sp. (Coleoptera: Ptiliidae), one of the smallest free-living insects. Arthropod Structure & Development 38: 247–270. 19101652 10.1016/j.asd.2008.11.003

[14] Polilov AA, Beutel RG (2010) Developmental stages of the hooded beetle *Sericoderus lateralis* (Coleoptera: Corylophidae) with comments on the phylogenetic position and effects of miniaturization. Arthropod Structure & Development 39: 52–69. 19835982 10.1016/j.asd.2009.08.005

[15] Beutel RG, Haas A (1998) Larval head morphology of *Hydroscapha natans* LeConte, 1874 (Coleoptera, Myxophaga, Hydroscaphidae) with special reference to miniaturization. Zoomorphology 18: 103–116.

[16] Grebennikov VV, Beutel RG (2002) Morphology of the minute larva of *Ptinella tenella*, with special reference to effects of miniaturisation and the systematic position of Ptiliidae (Coleoptera: Staphylinoidea). Arthropod Structure & Development 31: 157–172. 18088978 10.1016/s1467-8039(02)00022-1

[17] McKenna DD, Farrell BD (2010) 9-Genes Reinforce the Phylogeny of Holometabola and Yield Alternate Views on the Phylogenetic Placement of Strepsiptera. Plos One 5: e11887. 20686704 10.1371/journal.pone.0011887 PMC2912379

[18] Niehuis O, Hartig G, Grath S, Pohl H, Lehmann J, et al.. (2012) Genomic and morphological evidence converge to resolve the enigma of Strepsiptera. Current Biology (online early). 10.1016/j.cub.2012.05.018 22704986

[19] Pohl H (2010) A scanning electron microscopy specimen holder for viewing different angles of a single specimen. Microscopy Research and Technique 73: 1073–1076. 20196104 10.1002/jemt.20835

[20] Wipfler B, Machida R, Mueller B, Beutel RG (2011) On the head morphology of Grylloblattodea (Insecta) and the systematic position of the order, with a new nomenclature for the head muscles of Dicondylia. Systematic Entomology 36: 241–266.

[21] von Kéler SV (1963) Entomologisches Wörterbuch. Berlin: Akademieverlag. 679 p.

[22] Dybas HS (1976) The larval characters of featherwing and limulodid beetles and their family relationships in the Staphylinoidea (Coleoptera: Ptiliidae and Limulodidae). Fieldiana Zoology 70: 29–78.

[23] Selander RB (1982) Further studies of predation on meloid egg by meloid larvae (Coleoptera). Journal of the Kansas Entomological Society 55: 427–441.

[24] Selander RB (1987a) Behavioral observations in *Cyaneolytta* and a description of the triungulin larva of c-fryi (Coleoptera, Meloidae). Journal of the Kansas Entomological Society 60: 288–304.

[25] Selander RB (1987b) The type-species of *Zonitis* Fabricius and the synonymies of *Zonitis-flava* Fabricius and *Zonitis-ruficollis* Frivaldszky (Col-Meloidae). Deutsche Entomologische Zeitschrift 34: 341–350.

[26] Selander RB (1991) On the nomenclature and classification of the Meloidae (Coleoptera). Insecta Mundi 5: 65–94.

[27] Bologna MA (2003) *Australytta*, a new blister beetle genus from Southern Africa (Coleoptera: Meloidae). Annales de la Société Entomologique de France 39: 139–152.

[28] Bologna MA, Aloisi G (1992) Systematics of *Lydomorphus* Fairmaire 1882, with a description of the first instar larva of *L. dusaulti* (Coleoptera Meloidae). Tropical Zoology 5: 55–71.

[29] Bologna MA, Aloisi G (1994) Systematics and bionomics of *Physomeloe* Reitter, with description of the first instar larva (Coleoptera Meloidae). Eos-Revista Espanola de Entomologia 69: 45–56.

[30] Bologna MA, Pinto JD (1995) The triungulin of two Palaearctic *Meloe* subgenera: *Lasiomeloe* Reitter and *Micromeloe* Reitter (Coleoptera, Meloidae), with bionomic and taxonomic notes. Bollettino di Zoologia 62: 383–393.

[31] Bologna MA, Pinto JD (1998) A review of the Afrotropical species of *Meloe* (Coleoptera, Meloidae) with description of first instar larvae, a key to species and an annotated catalogue. Tropical Zoology 11: 19–59.

[32] Di Giulio A, Aberlenc HP, Taglianti AV, Bologna MA (2003) Definition and description of larval types of *Cyaneolytta* (Coleoptera Meloidae) and new records on their phoretic association with Carabidae (Coleoptera). Tropical Zoology 16: 165–187.

[33] Pinto JD, Bologna MA, Bouseman JK (1996) First-instar larvae, courtship and oviposition in *Eletica*: amending the definition of the Meloidae (Coleoptera: Tenebrionoidea). Systematic Entomology 21: 63–74.

[34] Turco F, Di Giulio A, Bologna MA (2006) First-instar larval morphology in the subtribe *Lydina* (Coleoptera, Meloidae, Lyttini), with discussion on its systematic value. Deutsche Entomologische Zeitschrift 53: 213–222.

[35] Bologna MA, Di Giulio A (2008) Revision of the genus *Trichomeloe* Reitter, with the description of new species and first instar larvae (Coleoptera: Meloidae). Contributions to Zoology 77: 227–248.

[36] Pinto JD, Selander RB (1970) The bionomics of blister beetles of the genus *Meloe* and a classification of the New World species. Illinois Biological Monograph 42: 1–222.

[37] Bologna MA, Fattorini S, Pinto JD (2001) Review of the primitive blister beetle genus *Iselma* with description of the first instar larva (Coleoptera, Tenebrionoidea). African Entomology 9: 105–129.

[38] Pohl H (2000) Die Primärlarven der Fächerflügler - evolutive Trends (Insecta, Strepsiptera). Kaupia 10: 1–144.

[39] Beutel RG (1995) Phylogenetic analysis of Elateriformia (Coleoptera: Polyphaga) based on larval characters. Journal of Zoological Systematics and Evolutionary Research 33: 145–171.

[40] Bologna MA, Pinto JD (2002) The Old World genera of Meloidae (Coleoptera): a key and synopsis. Journal of Natural History 36: 2013–2102.

[41] Bouchard P, Lawrence JF, Davies AE, Newton AF (2005) Synoptic classification of the world Tenebrionidae (Insecta : Coleoptera) with a review of family-group names. Annales Zoologici 55: 499–530.

[42] Young DK, Pollock D (1991) Description of the mature larva of *Pedilus flabellatus* (Coleoptera, Pyrochroidae, Pedilinae), with phylogenetic implications of the discovery. Canadian Journal of Zoology-Revue Canadienne de Zoologie 69: 2234–2238.

[43] Beutel RG (1999) Morphology and evolution of the larval head structures of Hydrophiloidea and Histeroidea (Coleoptera: Staphylinidae). Tijdschrift voor Entomologie 142: 9–30.

[44] Beutel RG, Hörnschemeyer T (2002a) Description of the larva of *Rhipsideigma raffrayi* (Coleoptera, Archostemata), with phylogenetic and functional implications. European Journal of Entomology 99: 53–66.

[45] Beutel RG, Hörnschemeyer T (2002b) Larval morphology and phylogenetic position of *Micromalthus debilis* LeConte (Coleoptera: Micromalthidae). Systematic Entomology 27: 169–190.

[46] Beutel RG, Ślipiński SA (2001) Comparative study of larval head structures of Sphindidae and Protocucujidae (Cucujoidea, Coleoptera). European Journal of Entomology 98: 219–232.

